# Supplementary material for: Investigating the neural correlates of smoking: Feasibility and results of combining electronic cigarettes with fMRI
Source: Sci Rep. 2017 Sep 12;7:11352. doi: 10.1038/s41598-017-11872-z (PMC5596056; doi:10.1038/s41598-017-11872-z)
Supplement: Supplementary file 1 — Supplementary Information [file 41598_2017_11872_MOESM1_ESM.pdf]

The effect of smoking on the brain revealed using electronic cigarettes with concurrent fMRI

Matthew B Wall*[a,b,c], Alexander Mentink[a,d], Georgina Lyons[e], Oliwia S Kowalczyk[e], Lysia Demetriou[a,b] and Rexford D Newbould[a,b].

[a]Imanova Centre for Imaging Sciences, Burlington Danes Building, Hammersmith Hospital, Du Cane Road, London, W12 0NN, UK
[b]Division of Brain Sciences, Imperial College London, Hammersmith Campus, Du Cane Road, London, UK
[c]Clinical Psychopharmacology Unit, University College London, 1-19 Torrington Place, London WC1E 7HB, UK.
[d]Leiden University, Rapenburg 70, 2311 EZ Leiden, The Netherlands
[e]Department of Psychology, Royal Holloway University of London, Egham, Surrey, TW20 0EX

***Corresponding Author:**

Matthew Wall
Imanova Centre for Imaging Sciences
Burlington Danes Building
Hammersmith Hospital
Du Cane Road
London, W12 0NN
United Kingdom
matt.wall@imanova.co.uk

Author Note
OS Kowalczyk's current affiliation is: Centre for Neuroimaging Sciences, Institute of Psychiatry, Psychology and Neuroscience, Kings College London, London, UK.

Supplementary Information

Methods

A custom-built opto-electronic device was used to record the light output of the LED of the ENDS during the scanning tasks. This consisted of a 10 metre optical fibre cable, attached to a small rubber connector that allowed it to be fitted to the ENDS, in the scanner room. The cable passed through a waveguide into the control room, and terminated in a light-tight box next to a photo-sensitive resistor. Wired in parallel with the resistor was a 1.5V power source (an AA battery) and a BNC cable. This circuit produced a voltage on the resistor whenever the ENDS was activated by an inhalation, and this time-series signal was recorded via the BNC connection by a standard analogue-to-digital data recorder (AD Instruments Powerlab). In this way compliance with the cued smoking task could be assessed, and the recorded data in the naturalistic smoking task could be used to define idiosyncratic 'smoking' events for each subject.

Results

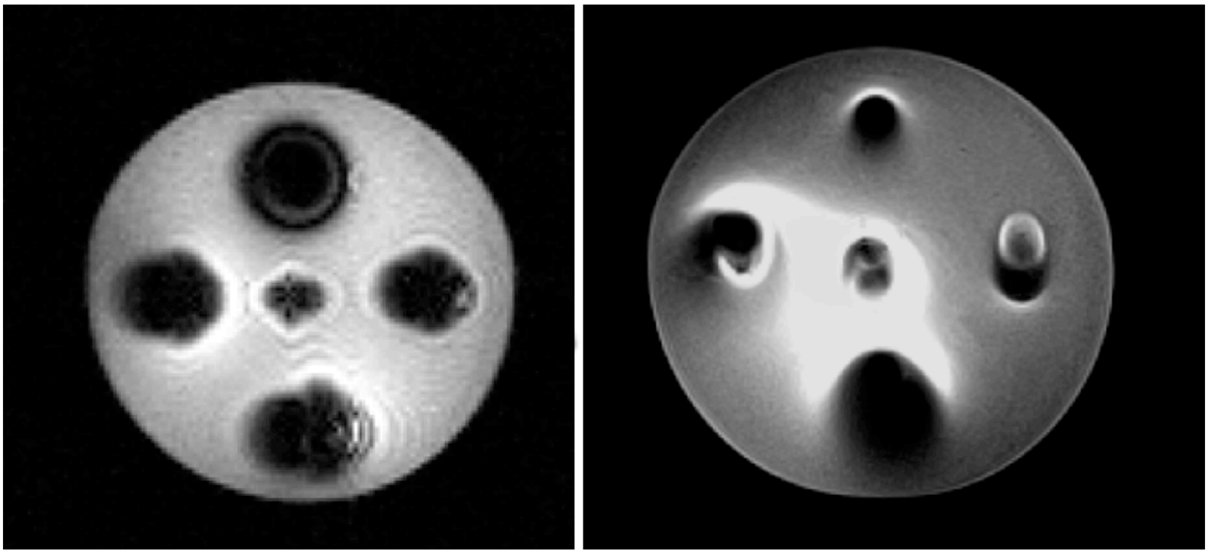

Figure S1. The five ENDS imaged with a gradient-echo sequence (left) shows characteristic dipole pattern disturbances from the different magnetic field susceptibilities found in the materials used to construct the varied ENDS. The same phantom images with a spin-echo sequence (right) removes the susceptibility-induced distortion and signal loss, but the RF field is compromised by the presence of conductors.

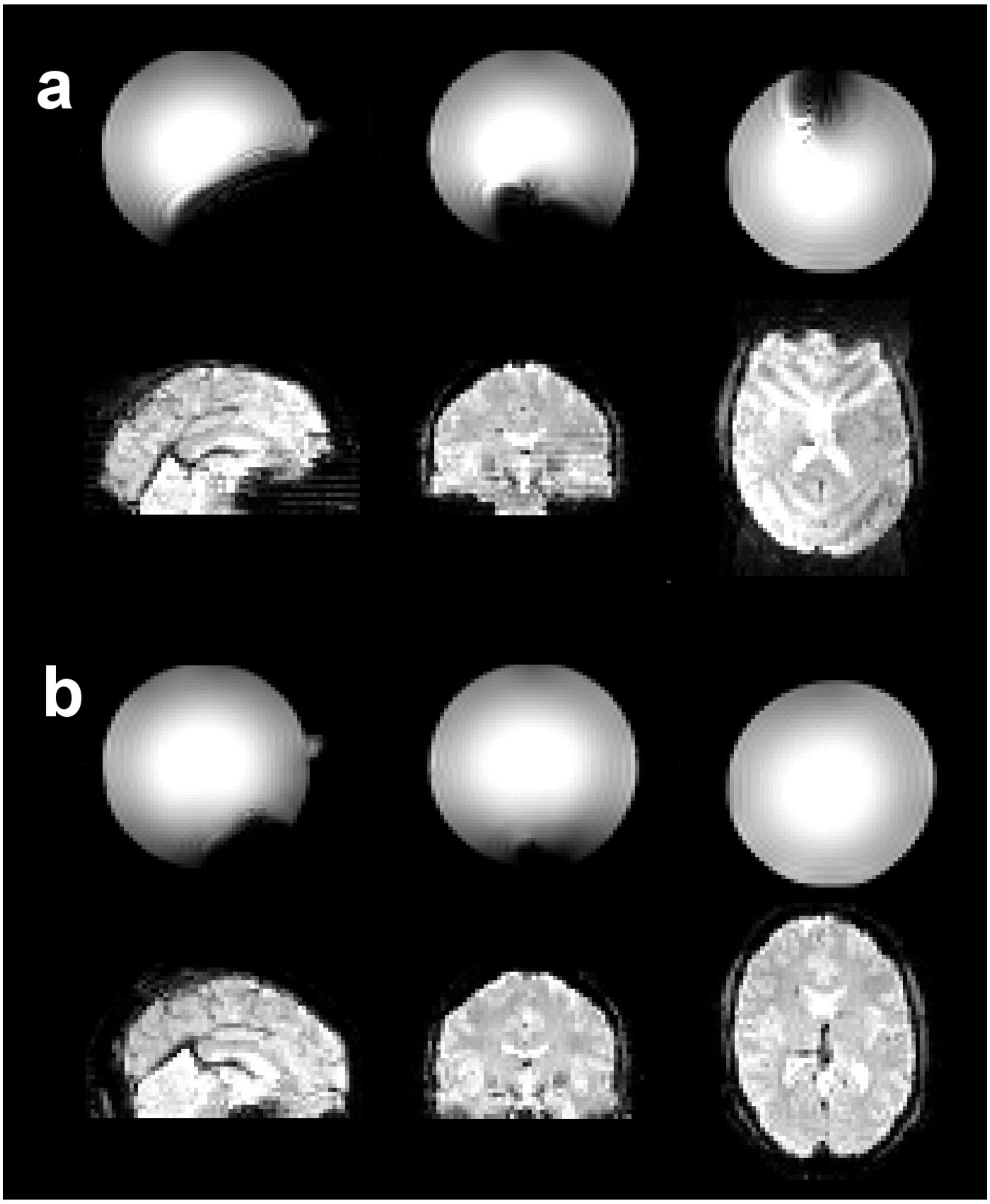

Figure S2. Results from initial testing of electronic cigarette products. a: An ENDS (‘Puritane’) with high magnetic susceptibility produces large regions of signal loss on a B0 magnitude image of an MRI phantom (top row). The same product also produces obvious artefacts on a BOLD EPI image when used by a human subject in the scanner (bottom row). b: A low magnetic susceptibility product (‘Njoy’) has much less effect on the B0 phantom image, and produces no obvious artefacts on BOLD EPI images from a human subject during use.

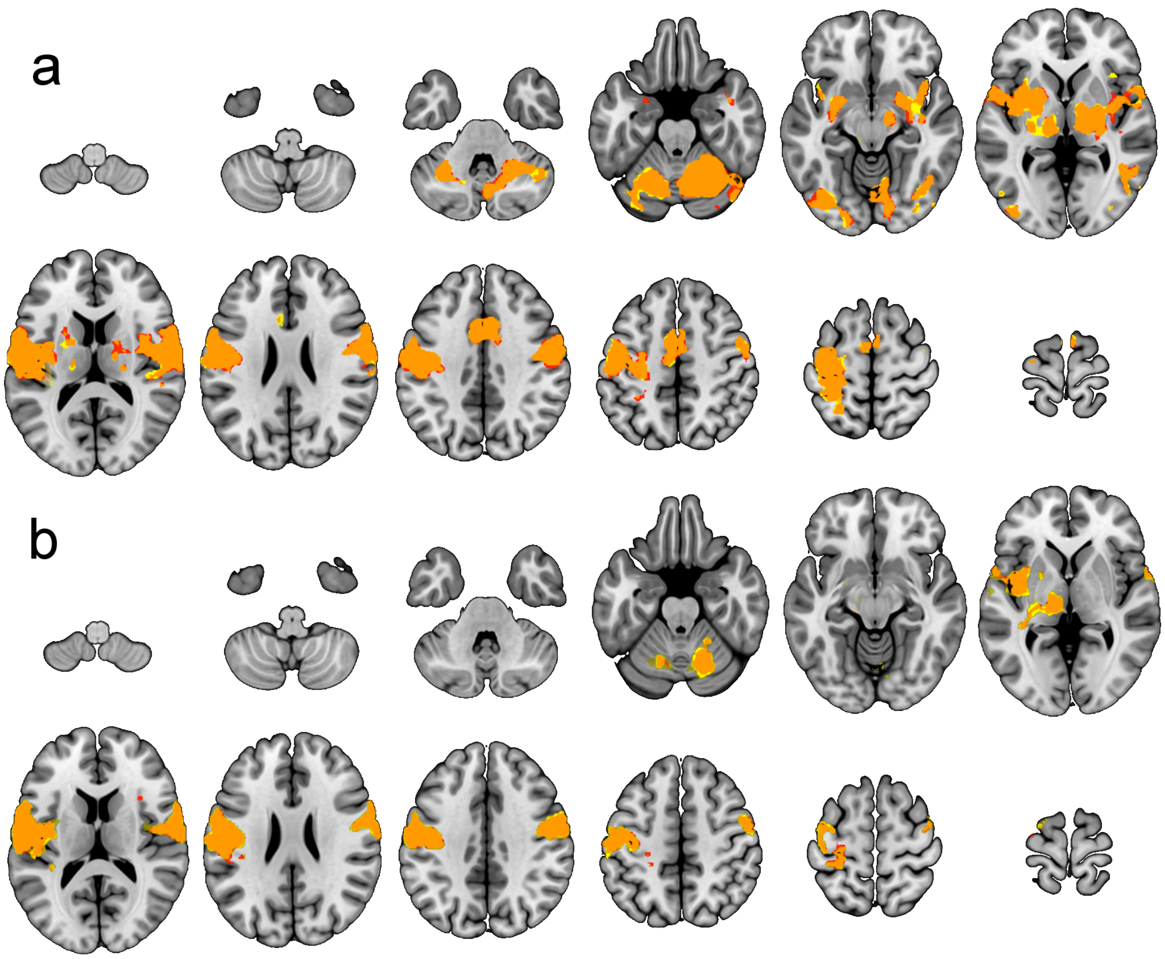

Figure S3. The effect of statistical modelling of physiological parameters (cardiac and respiratory effects) on the data from both experiments. Activation maps produced with no physiological modelling are shown in red, physiologically-corrected maps are shown in yellow, the overlap is in orange. a) Cued smoking experiment. b) Naturalistic smoking experiment. Effects of physiological noise modelling on the results are negligible, suggesting that these factors were not a significant confound in the data.
